# Very early onset dementias: Importance of differentiating from schizophrenia spectrum disorders

**DOI:** 10.1371/journal.pmen.0000107

**Published:** 2024-08-13

**Authors:** Lacey McCormick, Anu P. Bodla, Robert T. Rubin

**Affiliations:** 1 College of Osteopathic Medicine of the Pacific, Western University of Health Sciences, Pomona, California, United States of America; 2 Psychiatry Residency, Community Memorial Healthcare, Ventura, California, United States of America; 3 Department of Psychiatry and Biobehavioral Sciences, David Geffen School of Medicine at UCLA, Los Angeles, California, United States of America; PLOS: Public Library of Science, UNITED KINGDOM OF GREAT BRITAIN AND NORTHERN IRELAND

## Abstract

Very early onset dementias and other neurodegenerative diseases often present with prominent behavioral disturbances and can be initially misdiagnosed as schizophrenia spectrum disorders. Differentiating a primary psychiatric condition from a neurodegenerative cause is important, because there are considerable differences in prognosis, treatment, and the services required for effective management. To illustrate the implications of misdiagnosis, we provide case examples of very early onset dementias, most of which were initially diagnosed as schizophrenia or other psychotic disorder, owing to their unusually young age of onset and initial behavioral presentations. We suggest how a clinician can differentiate schizophrenia from rarer, early onset neurodegenerative causes of altered behavior and mentation, including behavioral variant frontotemporal dementia (bvFTD), Wilson’s disease, adult metachromatic leukodystrophy (MLD), Creuzfeldt-Jakob disease (CJD), and very early-onset Alzheimer’s disease. Schizophrenia with prominent obsessive-compulsive (OC) symptoms is briefly discussed, given that OC symptoms can be a major feature of dementias with prominent behavioral components.

## 1. Introduction

In this article we emphasize the importance of correctly differentiating very early onset dementias from schizophrenia spectrum disorders in young adults, in order implement appropriate management measures as soon as possible. The differential diagnosis of first-episode psychosis in a young person can be daunting. Initially, medical causes of psychosis, including substance use, infection, and metabolic disorders, must be ruled out before psychiatric disorders can be considered the primary cause. Challenges arise when patients do not fit the classic timeline or symptomatology of a schizophrenia spectrum disorder or do not improve with antipsychotic medication. Several neurological disorders with strong behavioral components can present in the same young age group. To illustrate the implications of misdiagnosis, we review several types of dementia in young persons that may present behaviorally similarly to schizophrenia and should be considered when the behavioral characteristics of the patient do not conform to the customary patterns of a schizophrenia spectrum disorder. The practical implications of misdiagnosis include multiple hospitalizations, distress to patient and family, and excessive use of healthcare resources, as exemplified in the following case reports of patients under age 30 with psychiatric symptoms at illness onset.

Table 1 summarizes cases demonstrating the prominence of behavioral disturbances occurring within several very early-onset dementias and neurocognitive diseases, including Behavioral Variant Frontotemporal Dementia, Wilson’s Disease, Adult Metachromatic Leukodystrophy, Creuzfeldt-Jakob Disease, and Very Early Onset Alzheimer Disease. Although cases presenting primarily with motor symptoms have been excluded, Wilson’s disease is included, owing to its often-significant neuropsychiatric presentation, which can dominate the clinical picture and lead to its misdiagnosis as a primary psychiatric disorder.

**Table 1:** Clinical characteristics of very early onset dementias.

Sex, Age at Present-ation (Years)	Initial Major Symptoms/ Signs	Initial Diagnosis	Symptom Progression	Time to Final Diagnosis	Determining Tests	Final Diagnosis	Time Initial Present-ation to Death	Refer-ence
Male 23	Flattened affect, stereotypies, behavioral disinhibition	Unspecified psychosis	Increased behavioral disinhibition, aggression	5 months	Brain CT, MRI	Behavioral Variant Frontotemporal Dementia	3 years	8
Female 29	Suicidal ideation, hyperphagia, sexual disinhibition	Schizophrenia	Behavioral changes, severe atrophy on MRI	5 years	Brain MRI, SPECT	Behavioral Variant Frontotemporal Dementia	---	9
Male 25	Social withdrawal, apathy, poor personal hygiene	Schizophrenia	Hallucinations, delusions, behavioral disinhibition, cognitive decline	5 years	Brain MRI, PET; brain biopsy	Behavioral Variant Frontotemporal Dementia	---	10
Female 17	Delusions, Auditory hallucinations, suicide attempt	Major depression with psychotic features	Continued psychotic symptoms, motor dysregulation	18 years	Ophthalmological exam, urinary copper, serum ceruloplasmin	Wilson’s Disease	18 years (suicide)	21
Male 13	Physically and verbally abusive, mood swings, slurred speech, clumsiness, nocturia	Adjustment reaction with mixed disturbance of emotion and conduct	Tearfulness, inappropriate laughter, temper tantrums, involuntary limb & head movements	5 years	Ophthalmological exam, head CT, urinary copper, serum ceruloplasmin & copper, liver biopsy	Wilson’s Disease	---	22
Male 18	Regressed behavior, auditory hallucinations, mutism	Acute psychotic reaction	Auditory hallucinations, delusions, labile affect, gait abnormality	12 years	Ophthalmological exam, serum ceruloplasmin, liver biopsy	Wilson’s Disease	---	23
Female 17*	Disorganized behavior, inappropriate laughter, auditory hallucinations, urinary incontinence	Schizophrenia	Attention deficit, slowed thought process, recent & remote memory disturbance	1 year	Brain CT, MRI	Metachromatic Leukodystrophy	---	26, 27
Female 25*	Depressed mood after childbirth	Postpartum depression	Cognitive decline, affective flattening, apathy, anhedonia	4 years	Neurological exam, brain MRI, neuropsychological testing	Metachromatic Leukodystrophy	---	26, 27
Male 40	Confusion, aggression, abnormal behavior	Differential included multiple psychiatric disorders	Labile affect, abnormal posturing, intrusiveness, hypervigilance, disorientation	3 years	EEG, brain SPECT, neuropsychological testing	Creuzfeldt-Jakob Disease	3 years	28
Male 19	Cognitive decline, impaired recall, memory disturbance	Schizophrenia prodrome	Increased recall, memory disturbance	3 years	Brain MRI, PET-CT, CSF biomarkers	Very Early Onset Alzheimer Disease	---	30

*These two cases are sisters.

We begin with brief reviews of dementia and schizophrenia, followed by case summaries that exemplify how the aforementioned illnesses can be misdiagnosed as primary psychotic disorders. We conclude with suggested diagnostic strategies to distinguish early onset dementias from schizophrenia spectrum disorders, and we emphasize the importance of correct diagnosis for treatment, illness prognosis, and support for patients and their caregivers.

## 2. Dementia

Dementia, now labeled major neurocognitive disorder in the Diagnostic and Statistical Manual of Mental Disorders, Fifth Edition (DSM-5) [1], indicates a decline in one or more cognitive domains severe enough to compromise social and/or occupational functioning. Cognitive domains that can be affected include complex attention, executive function, learning and memory, language, perceptual-motor ability, and social cognition. Clinicians use an array of psychological tests to identify dementia, ranging from brief screening instruments to complex, detailed neuropsychological assessments. The most common causes of dementia are Alzheimer’s disease, vascular dementia (from multiple, small strokes in the brain), frontotemporal dementia, and dementia with Lewy bodies (also seen in Parkinson’s disease). The multiple, other causes are less frequent [2]. The major dementias occur mainly in the elderly, with cognitive changes being the prominent symptom. However, the rare cases in late adolescence and young adulthood often present with prominent behavioral symptoms and thus can be easily misdiagnosed.

## 3. Schizophrenia

The DSM-5 criteria for schizophrenia include a history of impaired social and/or occupational functioning lasting six months or longer; at least one month of one positive symptom (delusions, hallucinations, or disorganized speech); and either a second positive symptom or additional disorganized behavior or negative symptoms (e.g., flat affect) [1]. Early-onset schizophrenia (ages 13–17) often presents with severe symptoms (auditory hallucinations, childhood-themed delusions, and flat or inappropriate affect) [3]. Deficits and delays in intellectual function can be prominent [4], along with other symptoms also occurring in early onset dementias (obsessive-compulsive symptoms, stereotypic mannerisms, highly disorganized behavior).

The peak age of schizophrenia onset is 21–25 years in men and 3–5 years later in women [5]. A typical reported case is a male college student, late teens or early 20s, with declining academic functioning, social withdrawal, and auditory hallucinations and/or delusional beliefs [6]. There is usually a 1–2 year prodromal period, with notable decline in function; e.g., in the context of education or employment [7]. Prodromal symptoms can include poverty of speech, apathy, social withdrawal, and decreased emotional expression. Early-onset dementias can mimic classic schizophrenia or have a strong psychiatric component at onset; e.g., in early-onset behavioral variant frontotemporal dementia (bvFTD), as discussed below.

## 4. Very early onset Behavioral Variant Frontal-Temporal Dementia (bvFTD)

There are few reported cases of very early onset bvFTD. We review three cases that illustrate different presentations, prompted by a patient in our hospital initially receiving a diagnosis of schizophrenia, which then proved to be very early-onset bvFTD [8]. We share the example of a 23-year-old man who presented with repetitive behaviors, motor apathy, flattened affect, behavioral disinhibition, and inappropriate laughter. A computerized tomography (CT) scan revealed generalized cerebral atrophy. He was admitted with a diagnosis of unspecified psychosis and discharged on antipsychotic medication. Three months later he was readmitted, diagnosed with schizophrenia, and again discharged on antipsychotic medication. Owing to symptom progression and aggressive behavior, he was readmitted two months later. CT scanning again revealed moderate central and cortical cerebral atrophy, and magnetic resonance imaging (MRI) showed severe, stable atrophy with frontotemporal predominance. He was diagnosed with probable bvFTD. Over the next year he rapidly deteriorated, and he died three years after his first admission [8].

Another case is a 29-year-old woman with a five-year history of treatment-resistant schizophrenia admitted for suicidal ideation, hyperphagia, and severe sexual disinhibition (asking strangers and family members to have sexual relations with her) [9]. Five years prior she had developed behavioral changes, including sexual disinhibition and wandering from home. Her mood and affect alternated between laughing and crying, she had feelings of anxiety and depression, and she complained of various somatic pains. In addition, she neglected personal hygiene, behaved compulsively by wetting her face throughout the day, and had a delusion of small lights being in her body, which remitted a year before admission. Her illness had not responded to antipsychotic, antidepressant, or antianxiety medication. On admission, she responded only with monosyllabic answers and “don’t know” replies, and she alternated between being fearful she was going to die and giggling inappropriately. She also had repetitive mannerisms such as humming and tapping her foot. Neurological examination, laboratory testing, and electroencephalogram (EEG) were within normal limits. However, brain MRI revealed bilateral, focal frontal and anterior temporal atrophy, and brain single photon emission computed tomography (SPECT) showed bifrontal hypoperfusion. She then was diagnosed with FTD and continued to deteriorate over the following two years.

In another example of misdiagnosis, Chu et al [10] reported a 25-year-old man presenting with increased introversion, social withdrawal, apathy, restlessness, lack of personal hygiene, and obsession with computer games. He was unresponsive to antidepressants. His restlessness and apathy worsened, and he became behaviorally disinhibited, prompting his first hospital admission at age 26. Abnormal behaviors included stealing from others and scratching strangers’ cars. He also experienced hallucinations and delusions of birds and of his family discarding him, leading to a diagnosis of schizophrenia–but he did not respond to antipsychotic medication. He denied history of drug use and had no family history of neurodegenerative disease. Over the next year he developed cognitive, executive, and memory dysfunction; however, neurological examination results were within normal limits. Brain MRI revealed bilateral decreased gray matter volume, and positron emission tomography (PET) indicated decreased metabolism in frontal, temporal, anterior cingulate, and subcortical areas. At age 28, he progressed with severe memory and cognitive decline, increased anger, and social disinhibition. By age 30, he was incontinent of urine and feces, bed-bound, and mute. He then was diagnosed with likely bvFTD. Brain biopsy performed three years after disease onset revealed mild cortical neuronal loss and shrinkage, with gliosis in the frontal lobe.

These examples illustrate how psychiatric signs and symptoms (psychosis, behavioral changes) in early onset bvFTD can result in an incorrect, initial diagnosis of schizophrenia and therefore suboptimal management. The presentation of early onset bvFTD includes behavioral and cognitive changes, disinhibition, aggression, apathy, hypersexuality/hyperorality (Klüver-Bucy syndrome), and compulsive behaviors [8–10]. Apathy, cognitive slowing, and unusual behavior also are components of schizophrenia, which complicates the differential diagnosis. Of importance, symptom progression over time and/or lack of response to psychotropic medication may point to a process other than schizophrenia.

Obsessive-compulsive (OC) symptoms such as compulsions and ritualistic behaviors can be prominent features of both FTD [11] and schizophrenia; about 25% of patients with schizophrenia have prominent OC symptoms [12]. OC symptoms also may be induced by second-generation antipsychotic medications [13]. OC symptoms, therefore, are not discriminatory for either diagnosis. For example, the patient in this case was obsessively counting car doors while driving, taking excessive daily showers, and ritualistically repeating the same daily routine. He incessantly repeated the same phrase and frequently inserted his thumbs into his nostrils, which he stated was to check if he was still breathing. Additional behaviors were standing on the sidewalk for hours, staring into neighbors’ windows, and responding to internal stimuli. Thus, OC symptoms are not specific towards one diagnosis and must be considered within each patient’s clinical context.

The Neuropsychiatric International Consortium for Frontotemporal Dementia advises next steps in the workup of primary psychiatric disorders vs FTD [14]. Because executive dysfunction occurs in both situations, repeated neuropsychological assessments can uncover progressive loss of function indicative of neurodegeneration. Brain imaging also can be repeated as symptoms progress, to demonstrate the presence of frontotemporal atrophy and hypometabolism, although not all FTD phenotypes show classic frontotemporal atrophy [14]. Genetic testing for common bvFTD variants and serum and cerebrospinal fluid (CSF) biomarkers can help differentiate bvFTD from other neurodegenerative processes. A detailed chart of family relationships also may prove helpful, owing to most patients with early onset bvFTD having a positive family history [15]. The correct diagnosis of bvFTD has major implications for prognosis and therapeutics [16], but there is no current treatment other than empirical symptom management [17].

## 5. Wilson’s disease

Wilson’s disease (hepatolenticular degeneration) is a rare, autosomal recessive disorder causing abnormal copper accumulation, particularly in the brain, cornea (Kaiser-Fleischer rings), and liver [18]. Presenting symptoms are varied. About 1/3 of patients present with neuropsychiatric symptoms and later develop hepatic (liver) symptoms [19]. Psychiatric presentations may include depression, bipolar, anxiety, and psychosis [18,20,21], possibly related to toxic copper deposition within the brain [22]. Early diagnosis and treatment can lessen and prevent irreversible neurological symptoms [20]; thus, differentiating the psychiatric symptoms of Wilson’s disease from those of a primary psychiatric disorder is imperative. The following three cases are illustrative of this:

A 35-year-old woman was hospitalized after a suicide attempt driven by persecutory and command auditory hallucinations [21]. She had involuntary jerking of all four limbs. Delayed development was observed at age 9, and at age 12 behavioral problems led to her expulsion from school. She experienced childhood seizures which were treated effectively. She had a prior suicide attempt at age 17, also owing to command auditory hallucinations, and had 11 prior psychiatric hospital admissions. On her last admission, Kayser–Fleischer rings (copper deposits around the iris of the eye) were noted. Total and direct bilirubin were slightly elevated, indicating early liver dysfunction. As well, serum ceruloplasmin and copper were low, and 24-hour urine copper was 240 μg (reference value < 80 μg; Wilson’s disease >100 μg). She was diagnosed with Wilson’s disease and penicillamine (copper-binding) treatment was initiated, but she died 11 days later from complications of her suicide attempt.

Another case is a 13-year-old boy with initial symptoms of physically and verbally abusing his siblings and stepmother [22], having experienced parental divorce, his father’s remarriage, and the birth of three stepsiblings. He developed inappropriate affect (laughing, crying), speaking to himself, clumsiness, mood swings, slurred speech, frequent nighttime urination, and he declined academically. On examination, he was noted to have mildly abnormal limb movements, Kayser-Fleischer rings, and liver and spleen enlargement. His EEG was normal, but head CT revealed diffuse brainstem and cerebral atrophy with localized low-density areas in the basal ganglia. A liver biopsy revealed an elevated copper concentration; serum copper and ceruloplasmin were low; and 24-hour urine copper was 304 μg. The patient was then diagnosed with Wilson’s disease, treated with trientine (copper-binding), referred to social work for help with family issues, and provided with special education addressing his cognitive deficits.

Saint Laurent [23] reported an 18-year-old man initially diagnosed with acute psychotic reaction. He presented with psychotic symptoms, including public nudity and auditory hallucinations. Antipsychotic treatment led to symptomatic improvement, and he was discharged after three months. Over the next five years, however, his social functioning declined, marked by academic failure and recurrent physical altercations, accompanied by insomnia, concentration difficulties, and weakness. He exhibited labile affect, auditory hallucinations, delusions of grandeur, and uncharacteristic hyper-religiosity, leading to a diagnosis of paranoid schizophrenia. Treatment with antipsychotic medication again was effective, although the patient also suffered from unexplained abdominal discomfort, nausea, and vomiting.

Over the next seven years he continued treatment with antipsychotic medication. He developed an abnormal gait, kyphosis, and joint flexion, requiring physiotherapy. At age 30, after his younger sibling’s diagnosis of Wilson’s disease, the patient was found to have Kayser-Fleischer rings and low serum ceruloplasmin. A liver biopsy was compatible with Wilson’s disease, and CT scan results were normal. Treatment for Wilson’s disease with penicillamine led to neuropsychiatric improvement, and antipsychotic medication was discontinued. However, it was re-started, owing to re-emergence of psychotic symptoms, but psychiatric management was more effective, with less medication required. At 15-month follow-up, the patient had sustained improvement with a combination of medications, although he still complained of intermittent abdominal pain and nausea.

This case highlights an initial presentation of psychosis in Wilson’s disease misdiagnosed as paranoid psychosis. Chelation (copper-binding) therapy partially improved the patient’s motor symptoms, and less antipsychotic medication was required for psychiatric management.

Adolescents and young adults often present with changes in personality, behavior, affect, and socialization, and initial laboratory studies may not be revealing [24]. These cases illustrate how Wilson’s disease may be diagnosed incorrectly as schizophrenia owing to suicide attempts, psychosis, and other behavioral manifestations. The first case [21] reflects one of the worst outcomes of delayed diagnosis, as the patient died from complications of her suicide attempt before she could receive effective treatment. Initial treatment with antipsychotic medication resulted in her developing tremors and severe akathisia, and her involuntary, choreiform movements, signs of Wilson’s disease, initially were considered a medication side effect.

A developmental history can be important in raising the suspicion of Wilson’s disease, although behavioral and developmental abnormalities in childhood can be confused with the schizophrenia prodrome. Specific examination and laboratory tests for Wilson’s disease are warranted in patients with treatment-resistant schizophrenia, history of jaundice, sensitivity to antipsychotic medication, and/or family history of neuropsychiatric disorders [21,22]. It is important to note that in Wilson’s disease, both cognitive and neurological symptoms can be mitigated with treatment, making the differentiation from a psychotic disorder even more critical. Correctly identifying and treating Wilson’s disease patients (e.g., copper chelation, liver transplantation) resolves or greatly lessens psychiatric symptoms and improves quality of life, usually leaving only mild cognitive changes that do not meet dementia criteria [19].

## 6. Adult Metachromatic Leukodystrophy (MLD)

MLD is a rare, autosomal recessive lysosomal disorder characterized by white matter demyelination in both the central and peripheral nervous system [25]. Behavioral abnormalities are the most common presenting symptom in adult MLD, which typically manifests in the late teens. Other symptoms and signs include dementia, psychosis, ataxia, polyneuropathy, and epileptic seizures [25]. Approximately 50 cases of confirmed adult MLD have been documented to date, and only a few have presented with schizophrenia-like symptoms. The following three cases are exemplary:

Two sisters with the psychocognitive form of adult MLD (prominent behavioral disturbance, mood changes, and progressive mental deterioration) had distinct presentations [26,27]. The younger sister, at age 17, developed disorganized behavior and inappropriate laughter. Over the next year, she withdrew socially and developed auditory hallucinations. Her school performance declined, and she became disoriented, culminating in psychiatric admission at age 18. On admission, she was disoriented to time, person, place, and identity. Her thought process was disorganized, her affect blunted, and her speech monotonous. She continued to experience auditory hallucinations and developed urinary incontinence and perceptual-motor difficulty. Routine laboratory studies were normal, but CSF protein concentration was elevated (a nonspecific indicator of central nervous system pathology). Given that the patient met criteria for schizophrenia, she was administered antipsychotic medication. Brain CT revealed cortical atrophy, ventricular enlargement, and white matter abnormalities; brain MRI showed diffuse white matter signal hyperintensity, especially in periventricular areas and the corpus callosum. Within four weeks of starting medication, her auditory hallucinations ceased, and psychotic symptoms diminished. Cognitive changes, including attention deficit, slowed thought process, and recent and remote memory disturbances, became apparent. Neuropsychological testing indicated a decline in full-scale intelligence quotient, in the range of severe intellectual disability. Impairments encompassed attention, perception, executive function, communication, and motor skills. Consequently, galantamine, an acetylcholinesterase inhibitor, was added to her treatment. Given her abnormalities on neurological examination, neuropsychological testing, and neuroimaging, an underlying neurological disorder was considered, and the patient was diagnosed with MLD, after testing for metabolic disorders showed reduced leukocyte arylsulfatase A activity and elevated urinary sulfatides (MLD markers).

In contrast, the patient’s older sister, age 25, presented with a milder, slower course of depression and negative symptoms of schizophrenia [26,27]. She had a postpartum depressive episode at age 21. At age 25, she showed decreased interest in her child and general lack of interest, with no other psychiatric symptoms, and was attending vocational school. At that time her younger sister was diagnosed with MLD, and testing led to a diagnosis of MLD in the older sister as well. At age 29, she was staying home, with a marked decrease in energy and interests. She had poverty of speech, increased response latency, affective flattening, apathy, anhedonia, social inattentiveness, and was unable to care for her child. She also showed decreased memory and attention. Brain MRI revealed symmetric enlargement of ventricles, bilateral frontal cortical atrophy, and periventricular, temporal subcortical and occipital subcortical white matter hyperintensities. As such, she was treated with galantamine.

Diagnosing adult MLD is challenging, owing to its rarity and varied behavioral and neurological manifestations. The above cases illustrate how MLD can present similarly to both the positive and the negative symptoms of schizophrenia. The younger sister presented with disorganized behavior, auditory hallucinations, and inappropriate laughter. In contrast, the older sister, following a prior postpartum depression, presented with decreased energy, flat affect, apathy, and social inattention. Because of their often-prominent behavioral features, it is important to consider metabolic disorders such as MLD in the differential diagnosis of schizophrenia with unusual characteristics and/or non-response to standard antipsychotic treatment.

## 7. Creuzfeldt-Jakob Disease (CJD)

CJD is a neurodegenerative disease caused by prion infection of the central nervous system. While CJD usually presents in later life, there are a few reports of onset at a younger age [28]. Psychiatric symptoms are more prevalent in these cases [29]: A 40-year-old man presented with progressive confusion and unusual, aggressive, and unpredictable behavior [28]. Initially noted by his wife were personality changes, including paranoia and irritability, leading to significant marital discord. He also manifested hypersexuality, visual hallucinations, a belief his body was numb, and restless sleep. His condition deteriorated, and he experienced daily headaches, mood swings, weight loss, and cognitive changes, including memory loss and difficulty concentrating. Extensive medical evaluation, blood studies, and neuroimaging (CT, MRI) initially revealed no significant findings. A SPECT scan, however, showed left frontal, left temporal, and biparietal hypoperfusion, and EEG showed diffuse slow wave abnormalities, consistent with an encephalopathy underlying his cognitive decline. Despite individual and marital counseling early in his illness and later treatment with antipsychotics, antidepressants, anti-anxiety agents, mood stabilizers, and electroconvulsive therapy, he showed only transient improvement. His condition continued to decline, leading to consideration of a neurodegenerative process of unknown etiology. He became mute, wheelchair-bound, and entered a nearly vegetative state, passing away within a year. Autopsy revealed extensive spongiform changes in his brain, consistent with CJD. Although CJD is a rapidly progressive and fatal disease, earlier diagnosis might have led to better palliative measures for this patient, with improvement in his quality of life.

## 8. Very early onset Alzheimer’s disease

Early onset Alzheimer’s disease (EOAD) is defined as Alzheimer’s disease in individuals under age 65 and often is genetically determined [30]. It manifests with progressive memory loss and other cognitive and behavioral symptoms. A case report of EOAD in a 19-year-old man, the youngest known case, indicates symptom overlap with those of schizophrenia [30].

At age 17 the patient reported decreased concentration, and at age 18 he reported difficulty reading and had decreased short-term memory. At presentation at age 19, he was living independently and showed no focal neurological signs. He had significant deficits in memory, including immediate recall, short-delay free recall (3 minutes later), and long-delay free recall (30 minutes later), and in long-delay recognition. Brain MRI revealed mild bilateral hippocampal atrophy, and brain PET-CT showed bilateral temporal cortex hypometabolism. CSF biomarkers indicated increased p-tau 181, decreased ⍺β_42/40_ ratio, and increased ⍺β_42_. Tests for autoimmune encephalitis, meningitis, and other metabolic, infectious, and toxic processes were negative. He was a carrier for the *APOE ε3/ε3* gene allele, but not for other clinically meaningful genetic variants.

We include this patient because his initial presentation of academic and cognitive decline in his late teens is similar to the schizophrenia prodrome. Clinicians should be open to alternative diagnoses of even extremely rare conditions if a patient continues to decline, as did this case, or does not respond to usual treatment.

## 9. Conclusions

Distinguishing early onset dementias from schizophrenia spectrum disorders poses a significant challenge, given the overlapping symptoms in these conditions [31]. Through case studies, we have illustrated how neurological disorders can present with prominent psychiatric symptoms resembling schizophrenia, potentially leading to misdiagnosis, delayed treatment, and consternation for patients and their families. In one study, 28% of patients diagnosed with a neurodegenerative disease had a prior incorrect psychiatric diagnosis [32]. It thus is important that clinicians be alert to patients who exhibit atypical behavioral and/or physical symptomatology or fail to respond to standard interventions. Correct diagnosis and prognosis may decrease psychiatric hospitalizations; e.g., the case of Wilson’s Disease reviewed above, with 11 readmissions [21], improve patients’ quality of life, and reduce inappropriate use of other healthcare resources. With regard to a progressively worsening dementia, informing the patient and family of the prognosis and likely functional decline can help preserve the safety of the patient and others from the future development of aggressive and other socially disruptive behaviors [8,29].

Comprehensive history taking is essential to an accurate diagnosis, especially developmental and family histories. Careful collateral history-taking from family members often can elucidate early symptoms, behavioral and otherwise, consistent with a schizophrenia prodrome vs. prominent, reduced cognitive abilities (memory, reasoning, etc.), as well as possible early motor changes, that can point to a very early onset dementia.

If a patient has affected relatives, genetic testing may be indicated [32]. Subtleties in symptomatology also are important; e.g., although ritualistic and compulsive behaviors can be a feature of bvFTD, a key difference between these behaviors in bvFTD vs. obsessive-compulsive disorder (OCD) is the lack of accompanying dysphoria in bvFTD. Complete neurological examination of course can reveal multiple findings indicative of an organic cause.

Neuropsychological testing, biomarker analysis, neuroimaging, and genetic testing all can aid in differential diagnosis. The Frontal Assessment Battery [33] and Addenbrooke’s Cognitive Examination [34] are tests that can aid in detecting early onset dementia. CSF analysis for amyloid and tau protein biomarkers can aid in diagnosis of early onset Alzheimer’s disease. Brain MRI at symptom onset may serve as a baseline image for patients who advance in symptoms or are nonresponsive to treatment [35]. In our case study of early-onset bvFTD presented above [8], the patient presented with generalized cerebral atrophy on CT; two months later, MRI revealed classic frontotemporal atrophy. A baseline MRI might have been revealing of frontotemporal changes. On the other hand, whether or not MRI should be part of the diagnostic workup in first-episode psychosis remains controversial, related, *inter alia*, to the low prevalence of findings that can guide treatment and the cost of MRI [36–40].

Physicians also face the challenge of differentiating symptoms of disease progression from medication-induced side effects; e.g., abnormal movements in Wilson’s disease [18] or OC symptoms resulting from second-generation antipsychotics [13]. An accurate timeline of symptom onset and improvement in relation to a detailed medication history can be helpful in these cases.

Finally, it should be noted that the diagnostic capabilities for early-onset dementias vary significantly across global healthcare systems. In resource-rich settings, advanced neuroimaging techniques, genetic testing, and comprehensive neuropsychological assessments may be readily available, facilitating more timely and accurate diagnosis. However, in resource-limited settings, access to such diagnostic tools may be constrained, leading to delays in correct diagnosis and suboptimal management. It is crucial for clinicians worldwide to be aware of these disparities and strive for accurate diagnosis through available means, while advocating for improved diagnostic resources and training in under-resourced regions.

In summary, atypical presentations of schizophrenia and/or lack of a robust response to medication may indicate a rare, early onset neurodegenerative process presenting with psychiatric symptoms. In addition to standard diagnostic procedures, extensive cognitive neuropsychological testing, neuroimaging (CT, MRI, PET), and analysis of CSF biomarkers often are needed to substantiate a diagnosis of early onset dementia [35]. The course and treatment requirements of a primary psychotic illness vs. an early onset dementia in a person of similar age are different, as are the medical resources needed to effectively manage each type of illness. Early diagnosis, therefore, is essential for initiating appropriate treatment, anticipating a potentially poor prognosis, and providing the supportive services that protect the well-being of both patients and those around them.
